# Supplementary material for: Profiling Epigenetic Aging at Cell‐Type Resolution Through Long‐Read Sequencing
Source: Aging Cell. 2025 Jul 2;24(8):e70084. doi: 10.1111/acel.70084 (PMC12341782; doi:10.1111/acel.70084)
Supplement: Supplementary file 1 — Figure S1. [file ACEL-24-e70084-s001.pdf]

Supplementary Figures:

Profiling epigenetic aging at cell-type resolution through long-read sequencing

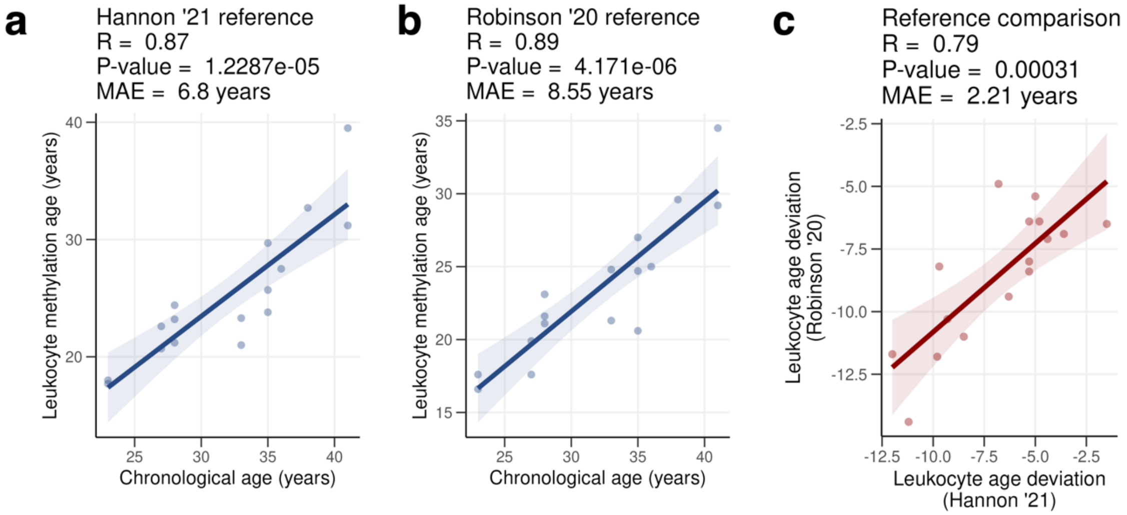

Supplementary Figure 1: Robustness of LongReadAge to reference dataset. Application of LongReadAge to 16 long-read leukocyte samples with Hannon ’21 reference **(a)** and Robinson ’20 reference **(b)**. **(c)** Comparison of age deviation (predicted minus actual age) between Hannon ’21 and Robinson ’20 reference datasets for the same leukocyte long-read dataset.

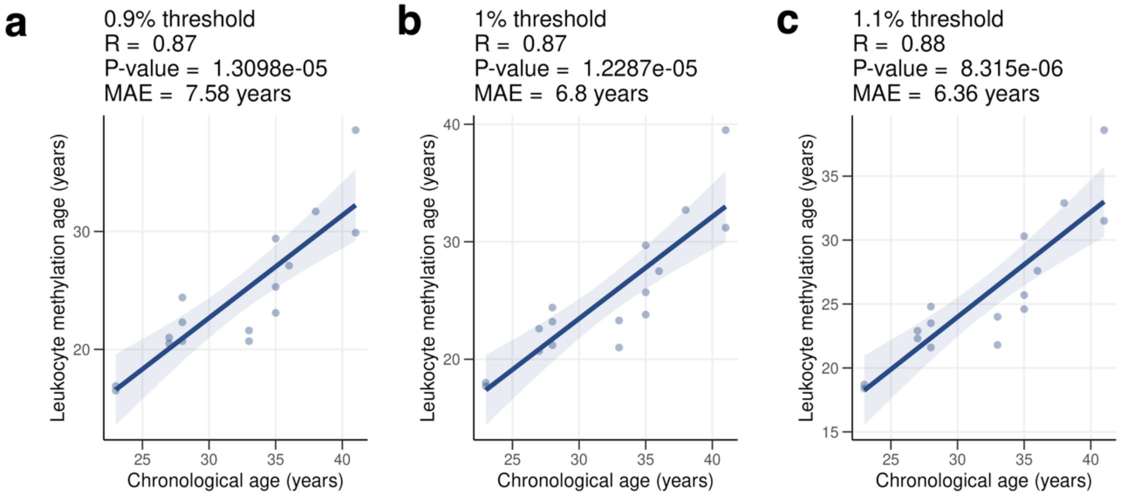

Supplementary Figure 2: Robustness of LongReadAge to methylation site threshold. Application of LongReadAge to 16 long-read leukocyte samples with Hannon ’21 reference and three percentile cutoffs for selecting top age-correlating CpG sites: 0.9% **(a)**, 1.0% **(b)**, and 1.1% **(c)**.

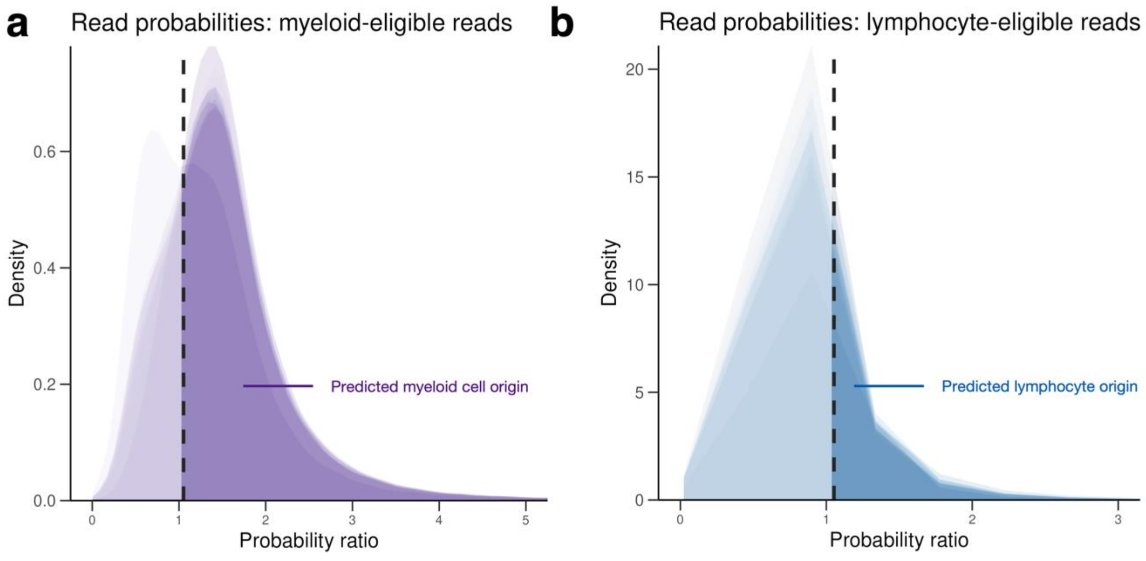

Supplementary Figure 3: Single molecule read probability distributions. Distribution of cell-type-origin probability ratios for single-molecule long-reads overlapping with myeloid cell-specific differentially methylated regions **(a)** and lymphocyte-specific differentially methylated regions **(b)** for all 16 leukocyte samples. Reads with a probability ratio exceeding 1.05x are classified as myeloid cell-originating or lymphocyte-originating based on concordance with a reference cell-type methylome.

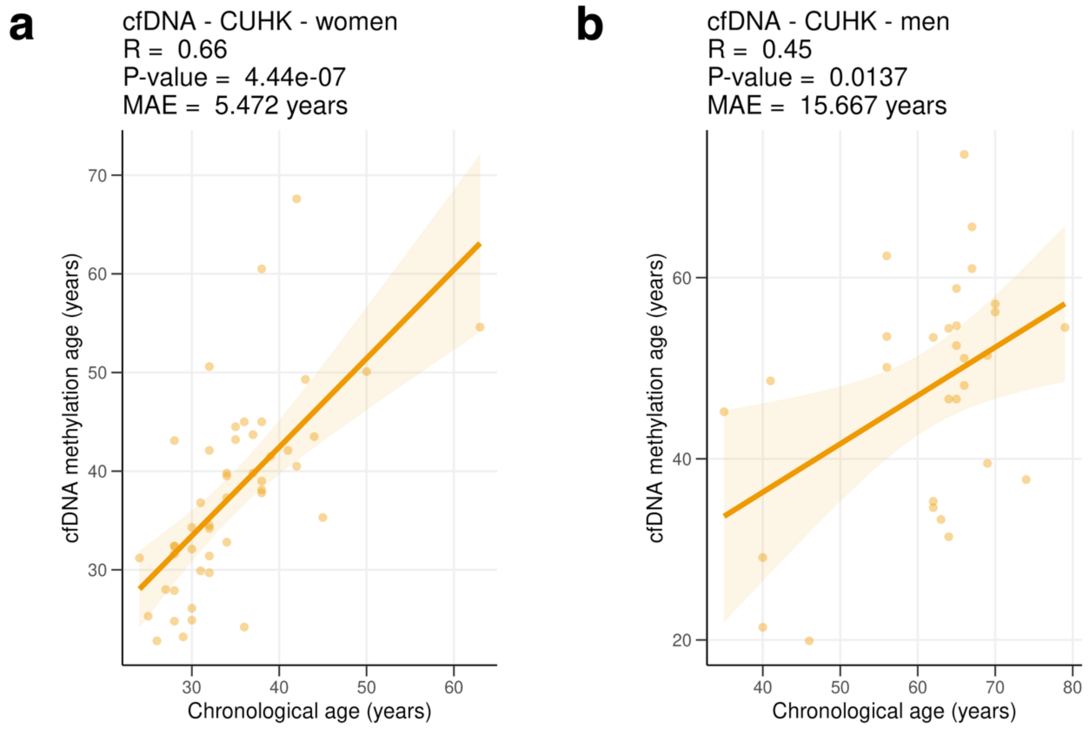

Supplementary Figure 4: Sex-specific CUHK cfDNA accuracy metrics. Application of LongReadAge to CUHK cohort for women **(a)** and men **(b)** separately.
